# Trial for Manslaughter—Medical Testimony

**Published:** 1854-09

**Authors:** 


### TRIAL FOR MANSLAUGHTER—MEDICAL TESTIMONY.

WE copy the following report of a trial for Manslaughter from the *Boston Medical and Surgical Journal.* The trial occurred at Janesville, Wisconsin, and is of a character to interest our readers independently of the part performed in it by members of the medical profession; but taken in connection with the testimony of some of the medical witnesses, it is certainly one of the most curious on record. That such testimony should be given in court by physicians here in the middle of the nineteenth century, may well excite our wonder. Our medical colleges and medical associations, we fear, will not reach such cases as those of Dr. Grafton, Dr. Ball, and Dr. Lewis:—

"The following case may prove interesting to the profession, as showing, to some extent, the kind of medical testimony upon which criminal prosecutions are sometimes founded.

"On the 26th day of July last, Miss E. M. Smith, a beautiful and intelligent young lady, who is engaged in teaching a school in this city, was arrested and brought before Justice Bates, on a charge of manslaughter. Upon the examination it appeared that upon the 22d day of June, Miss S. had punished a boy, (9 or 10 years old) by whipping him with a green switch, about half an inch in diameter at the butt, some two feet long, and tapering off to a point; and that during the whipping, some three or four blows fell upon the head, two of which raised small stripes or welts, which remained for some four or five days. The teacher was represented as having corrected the boy not in an angry or excited manner, but properly and mildly. No unusual effects were observed upon the scholar at the time. He remained until the close of the school, and spelt in his class as usual. In the afternoon he complained of some headache, and on the next day (the 23d) he vomited and bled at the nose. On the 24th he was at school again. At night he complained of some headache and nausea, and was quite restless. On the 25th he took some patent pills, but got no benefit from them. On the 26th he was confined to the bed, and on the 27th Dr. Grafton was called to see him, and continued in attendance until his death, which took place July 7th. This is the substance of the non-professional testimony in the case. For the prosecution, Drs. Grafton, Ball, Lewis, and Wood, were called; and for the defense, Drs. Mack, Chittenden, Barrows and Pease. We will give a brief synopsis of the testimony of these gentlemen. Those portions of the testimony of Drs. Grafton and Mack, which are italicised, are to be compared with each other, that their disagreement may be fully noted.

*Dr. Grafton sworn*—Was the attending physican in this case. Called first, June 27th. Patient had no high favor at any time. Saw nothing resembling fever. Skin usually of natural temperature; often cool and sweaty. Pulse at no time frequent and strong; towards the last frequent and feeble. Tongue had a whitish, or light yellow coat. Bowels rather costive. Vomitting once or twice after he saw him. A little delirious once or twice when roused to take a drink or medicine. This was only momentary. July 2d had a slight spasm of the arms and legs, which he (Dr. G.) attributed to irritation from operation of physic. No other convulsions at any time. Eyes never suffused or injected. Thought the pupils at one time not so large as they should be, considering the light in the room. Eyes were sensative to light, and he complained of noise hurting him. Had violent paroxysms of pain in ears and eyes; face would flush, and he would cry out, "Oh my eyes! oh my eyes!" Began to sink into a comatose state on the 5th, and died comatose on the 7th, at 8½, P. M. Conducted the post mortem. No evidences of external injury. *No passive conjestion about the brain. Membranes injected with arterial blood.* Some gelatinous effusion upon the convolutions of the brain. Membranes adherent to the brain at a point on the top of the head, an half inch by two or two and a half inches in size. *Arachnoid thickened at the base of the brain. Adherent to pia mater on the surface of the hemisphere. Six or eight ounces of turbid effusion in the ventricles. Some flocculi of lymph in fourth venticle. No tubercles in the lungs. Mucous coat of stomach and bowels perfectly healthy. Considered the case one of well-marked inflammation of the membranes of the brain, affecting their entire substance. Symptoms were such as would not be present in any other disease. Pathological appearances unmistakable. The gelatinous effusion could not be produced except by inflammation. Believed the whipping was*

*the exciting cause of the disease; that it must have operated by producing concussion of the brain.* Made up his opinion that the blows were the cause of the disease, *from the absence of all other causes in this case. Thinks he could have detected any other causes if they had been present.*

*Dr. Ball sworn*—Has heard the testimony in the case. Has no doubt that the boy died of inflammation of the brain produced by the whipping. Whipping produced concussion on the brain. *Cross-examined*—Been practising some twenty years. Been to California. Is now improving a farm. Preaches some on Sundays. Has no doubt that the blows produced concussion on the brain. Counsel ask—What is concussion of the brain? Witness hesitated a long time, and at last, when the question was repeated, "they must excuse him, he was so confused he could not tell."

*Dr. Lewis sworn*—Was present at the post-mortem examination. Thinks the case was one of inflammation of the brain, or hydrocephalus. Thinks the blows were the *remote* cause of the disease. Congestion of the brain was arterial. *Cross-examined*—Arterial congestion all over the brain. Could not tell in what membranes. Could not tell what vessels were congested. Afterwards said he could tell, but would not. Is a regular practising physician. No boy's business whether he ever graduated or not. Finally, said he had graduated at Eairfield. Did not receive a diploma. Students often graduate without receiving diplomas. Counsel here inquired if there were not two methods of graduating, one with white and the other with black balls, and if it was not in the latter method that no diplomas were given?

*Dr. Wood sworn*—Has heard Dr. Grafton's testimony, and fully concurs with him in his views in relation to this case.

DEFENSE. *Dr. Mark sworn*—Was consulting physician this Had disagreed entirely with Dr. Grafton in their consultations. Considered the cause to have been one of bilious remittent fever of a low type. Had advised a gentle tonic plan of treatment. Patient at one time took thirty-five grains of quinine, by his advice, to arrest a chill that was expected in the morning; thought the patient was decidedly better after taking the quinine. Dr. Grafton refused to continue the tonic and sustaining course of treatment after the quinine was given, but placed the boy upon the use of calomel, morphine, croton oil, digitalis, antimony, wine of cochicum and nitre. He continued this course until the day before the patient's death. Believes these articles were highly injurious, the disease being one of a low grade of action, with no time any high vascular excitement. Thinks the patient died of passive congestion of the brain, supervening upon a state of debility; that the persistent use of opium, digitalis and tartarized antimony, had directly tended to this result. Was present at the post-mortem examination. Meningeal vessels deeply congested with *dark venus blood.* Slight gelatinous or jelly-like effusion between the convulsions. Considered this the result of the simple congestion alone. *Might be produced by inflammation, but by itself was not positive evidence thereof.* Thought the small spot of adhesion of the dura mater on the top of the brain not recent. Judged by its firmness, and the absence of any effusion or discoloration in its vicinity. There was no plastic or organized lymph, or liquid or concrete pus found at any time during the examination. *There was no abnormal thickening or adhesion of the arachnoid.* This membrane is thicker at the base of the brain, and it is adherent by fine cellular substance to the pia mater over the surface of the hemispheres. Fluid in the ventricles was allowed to escape and run out upon the table, mixing with the blood, *so that it was impossibce to determine its character or quantity. Saw no flocculi of lymph in the fourth ventricle. The lungs were not examined. Saw nothing at the post-mortem examination, and had heard no testimony during this trial, that would justify him in attributing, in any way, this boy's sickness and death to the whipping he had received.* Inflammation of the brain from external injury would occur directly, in one of two ways. Either traumatic inflammation originated at the point of injury and traceable directly thereto, or inflammation ensuing upon the re-action from concussion, frequently originating at a point remote from the seat of violence, and exhibiting upon a post mortem examination no pathological connection between the external injury and the inflammation which had followed it. In these cases, the fact that the brain had suffered from concussive force, must be shown from the character of the symptoms which followed the infliction of the blows. *In the case now under consideration there were none of the symptoms of concussion of the brain described as having been present at the time of the whipping, and in fact, the character of the whip used was such as to preclude the possibility of any such effect having been produced.* Even if this case had been (as Dr. Grafton assumes) one of simple acute meningitis, *still no man could honestly and intelligibly testify to the absence of all other causes except the whipping to have produced it*, as there are some causes of this disease which are wholly unknown, and the presence of which, heretofore, *even Dr. Grafton himself would not be able to detect.*

Drs. Chittenden, Barrows and Pease, were sworn. Were all present at the post-mortem examiuation. They coincided with Dr. Mack in his description of the appearances observed, and sustained him in his views in relation to the case.

This closed the defense. The Justice promptly dismissed the complaint, and the citizens presented Miss Smith with a beautiful gold watch and chain as an evidence of their sympathy for her unjust and malicious *persecution.*

